# Household responses to malaria: illness perception, cost implications and treatment-seeking behavior of mothers in Calabar, Nigeria

**DOI:** 10.1186/1475-2875-13-S1-P102

**Published:** 2014-09-22

**Authors:** Susan Etim, Jude Ogbeche

**Affiliations:** 1 University of Calabar, Calabar, Nigeria

## 

Malaria contributes substantially to the poor health situation in sub-Saharan Africa accounting for over 2.5 million deaths annually. About 50% of malaria cases present as outpatient visits and 20% as hospital admissions, while 30% of the suspected malaria cases are managed at home using traditional remedies and drugs bought from the local chemist. Malaria, as a serious public health problem, has severe consequences on people of all ages with the greatest burden on the world's poorest communities by affecting productivity and household income. Vulnerability to the consequences of malaria has a lot to do with problems of poverty, access to and cost of treatment and prevention. These are strongly correlated with household income and socio-economic status. There is limited information on the household cost implications and treatment-seeking behavior especially among the urban poor. There is also a need for a better understanding of the malaria infection and disease process to guide the design of effective policies and tools, acceptable and affordable to the people in an attempt the reduce the malaria problem. This paper will examine and determine the factors that influence the perception of malaria, cost implications for treatment and treatment-seeking behavior and preventive measures among the urban poor mothers in Calabar, Nigeria.

